# A Four-Antigen Mixture for Rapid Assessment of *Onchocerca volvulus* Infection

**DOI:** 10.1371/journal.pntd.0000438

**Published:** 2009-05-19

**Authors:** Peter D. Burbelo, Hannah P. Leahy, Michael J. Iadarola, Thomas B. Nutman

**Affiliations:** 1 Neurobiology and Pain Therapeutics Section, Laboratory of Sensory Biology, National Institute of Dental and Craniofacial Research, National Institutes of Health, Bethesda, Maryland, United States of America; 2 Laboratory of Parasitic Diseases, National Institutes of Health, Bethesda, National Institutes of Health, Bethesda, Maryland, United States of America; The University of Nottingham, United Kingdom

## Abstract

**Background:**

Onchocerciasis, an infection caused by the filarial nematode *Onchocerca volvulus*, is a major public health concern. Given the debilitating symptoms associated with onchocerciasis and concerns about recrudescence in areas of previous onchocerciasis control, more efficient tools are needed for diagnosis and monitoring of control measures. We investigated whether luciferase immunoprecipitation systems (LIPS) may be used as a more rapid, specific, and standardized diagnostic assay for *Onchocerca volvulus* infection.

**Methods:**

Four recombinantly produced *Onchocerca volvulus* antigens (Ov-FAR-1, Ov-API-1, Ov-MSA-1 and Ov-CPI-1) were tested by LIPS on a large cohort of blinded sera comprised of both uninfected controls and patients with a proven parasitic infection including *Onchocerca volvulus* (*Ov*), *Wuchereria bancrofti* (*Wb*), *Loa loa* (*Ll*), *Strongyloides stercoralis* (*Ss*), and with other potentially cross-reactive infections. In addition to testing all four *Ov* antigens separately, a mixture that tested all four antigens simultaneously was evaluated in the standard 2-hour incubation format as well as in a 15-minute rapid LIPS format.

**Findings:**

Antibody responses to the four different *Ov* antigens allowed for unequivocal differentiation between *Ov*-infected and uninfected control sera with 100% sensitivity and 100% specificity. Analysis of the antibody titers to each of these four antigens in individual *Ov*-infected sera revealed that they were markedly different and did not correlate (*r_S_* = –0.11 to 0.58; *P* = 0.001 to 0.89) to each other. Compared to *Ov*-infected sera, patients infected with *Wb*, *Ll*, *Ss*, and other conditions had markedly lower geometric mean antibody titers to each of the *Ov* 4 antigens (*P*<0.0002 for each antigen). The simplified method of using a mixture of the 4 *Ov* antigens simultaneously in the standard format or a quick 15-minute format (QLIPS) showed 100% sensitivity and 100% specificity in distinguishing the *Ov*-infected sera from the uninfected control sera. Finally, the QLIPS format had the best performance with 100% sensitivity and specificity values of 76%, 84% and 93% for distinguishing *Ov* from *Wb*, *Ll* and *Ss*-infected sera.

**Conclusions:**

The multi-antigen LIPS assay can be used as a rapid, high throughput, and specific tool to not only to diagnose individual *Ov* infections but also as a sensitive and potentially point-of-care method for early detection of recrudescent infections in areas under control and for mapping new areas of transmission of *Ov* infection.

## Author Summary

Caused by the filarial parasite *Onchocerca volvulus*, onchocerciasis is a neglected tropical disease associated with blindness and severe dermatitis. Available diagnostic methods are either invasive, require hours or days to perform, and/or need sophisticated equipment to be conducted. Thus, there is an urgent need for simple and rapid technologies for the specific diagnosis of *Onchocerca volvulus* infection. Here we investigated whether luciferase immunoprecipitation systems (LIPS) can produce a more rapid and specific test for diagnosis of *O. volvulus* infection. Using modified versions of previously identified *Onchocerca*-specific antigens, LIPS tests detected antibodies to all four *O. volvulus* antigens and easily distinguished the *O. volvulus*-infected samples from uninfected samples. We also tested these four different antigens in a simpler format as a combined mixture and distinguished 100% of the confirmed cases from the uninfected controls. A rapid 15-minute version of this mixture test (QLIPS) also allowed distinction of 100% of the cases from those uninfected and performed even better in identifying *Onchocerca* from other cross-reactive parasitic infections. This study suggests that this rapid LIPS test (QLIPS) has the potential to be used in point-of-care detection of onchocerciasis and thereby may provide a new tool for diagnosis and the monitoring of transmission control measures.

## Introduction

As one of the neglected tropical diseases (NTDs), onchocerciasis (or ‘river blindness’), caused by the filarial parasite *Onchocerca volvulus* (*Ov*), can lead to blindness and disabling dermatitis. Past and ongoing control measures, aimed at interrupting transmission by vector control (Onchocerciasis Control Programme in West Africa, OCP), reducing the burden of morbidity to tolerable levels (African Programme for Onchocerciasis Control, APOC), and eliminating the reservoir of infection wherever possible (Onchocerciasis Elimination Program for the Americas, OEPA), have led to substantial decreases in the prevalence of infection and the risk of blindness [1]. Despite these measures, which currently rely almost exclusively on ivermectin distribution, estimates suggest that 37 million people remain infected with *Ov* with an additional 90 million people being at risk in Africa [2]. Superimposed on this estimate of *Ov*-infected individuals has been the concern about ivermectin resistance [3] and the serious adverse events associated with ivermectin administration in areas where another filarial parasite, *Loa loa*, is co-endemic [4].

In support of elimination programs for onchocerciasis, various criteria for elimination have been proposed that rely on sensitive molecular xenomonitoring of infection in the *Simulium* vectors, epidemiologic and clinical criteria, and proven diagnostic assessments [5]. For the diagnostics in support of certification programs for onchocerciasis elimination, detection of microfilariae in skin snips have long held primacy, although sensitive and specific serodiagnostic assays [6] have largely supplanted skin snipping because these antibody-based tests are less invasive, more sensitive and can detect pre-patent infection [7]. A variety of serological tests employing different *Ov* antigens have been described (reviewed in [8] including those that have used cocktails of antigens [9],[10],[11]. Each antigen, when tested, has had the characteristic of identifying *Ov* infection early (often pre-patency) in the infection. More recently a field-applicable diagnostic immunoassay based on one of these Ov-specific recombinant antigens, Ov-16, showed 80% sensitivity for detecting *Ov*-infected sera [12],[13], while an ELISA employing a recombinant hybrid *Ov* protein showed 93% sensitivity [14],[15]. Despite the high sensitivity of all these immunoassays, each of these tests have had some difficulty discriminating *Ov*-infected sera from some other filarial infections that can be co-endemic with *O. volvulus* such as *Wuchereria bancrofti* (a causal agent of lymphatic filariasis) and *L. loa* (the causal agent of loiasis).

Recently, *Renilla* luciferase (Ruc)-antigen fusions produced in Cos1 cells were used in a simple immunoprecipitation assay called LIPS (denoting luciferase immunoprecipitation systems) to measure antibody responses to infections by the intestinal nematode *Strongyloides stercoralis* (*Ss*) [16] and the filarial nematode *L. loa* (*Ll*) [17]. In these studies, LIPS showed improved performance to existing ELISAs and offered a highly sensitive, robust and high-throughput testing format. In the present study, we utilized the LIPS technology for the assessment of Ov-specific antibodies. The results presented here demonstrate that LIPS assay detection of antibodies to a four-antigen cocktail in a standard 2-hour format or with a rapid 15-minute LIPS test (so-called QLIPS, for quick LIPS) generates a highly robust, sensitive and specific test for identifying *O. volvulus* infection.

## Materials and Methods

### Ethics statement

Informed written consent was obtained from all subjects in accordance with the human experimentation guidelines of the Department of Health and Human Services under multiple NIAID IRB-approved protocols, and the studies were conducted according to the principles expressed in the Declaration of Helsinki. All patient identification codes have been removed in this publication.

### Human sera

For the present study, great care was taken to choose sera from areas where there was no overlap between onchocerciasis and other filarial infections. Thus, pre-treatment sera were taken from well-characterized (*O. volvulus* microfilaria-positive, MF+) patients with onchocerciasis from Ecuador and Guatemala [18]. Sera from patients with documented *Wuchereria bancrofti* (*W. bancrofti* MF+ and circulating filarial antigen positive) were obtained from India, Guyana, the Comoros Islands and the Cook Islands, those with loiasis (*L. loa* MF+) from an area of Benin where there is no or *W. bancrofti* or *O. volvulus* [18], and those with strongyloidiasis (*S. stercoralis* larvae in fecal samples) [16] from Southeast Asia. Additional sera came from well-characterized patients seen by the Clinical Parasitology Unit, Laboratory of Parasitic Diseases, National Institute of Allergy and Infectious Diseases, National Institutes of Health. Some of these sera included four with Hyper-IgE syndrome (HIE), four with Hypereosinophilic syndrome (HES), as well as four others with non-filarial parasitic infections. Control uninfected sera came from North American subjects with no history of exposure to filarial or other nematode parasites and who had never traveled out of North America. A more detailed summary of the patient sera used is shown in Table 1.

**Table 1:** Patient population for serologic studies.

Group	Source of population	No.
Control	United States	72
Onchocerciasis (MF+)	Guatemala	11
	Ecuador	21
	Cameroon*	4
	Sierra Leone	2
Loiasis (MF+)	Benin	90
*Wuchereria bancrofti* (MF+)	Cook Islands	3
	Comoros Islands	2
	Guyana	1
	India	84
*Strongyloides stercoralis*	SE Asia	27
Miscellaneous	Hyper-IgE syndrome (HIE)	4
	Hypereosinophilic syndrome (HES)	4
	Non-filarial parasitic infections	4

*: Negative for daytime microfilariae of *Loa loa* and negative for *W. bancrofti* circulating filarial antigen.

### Generation of Ruc-antigen fusion constructs

A mammalian *Renilla* luciferase (Ruc) expression vector, pREN2, was used to generate all plasmids [19],[20]. The four *Ov* antigens used in the LIPS assays included fatty-acid and retinol-binding protein-1, Ov-Far-1/Ov-20 [21]; aspartyl protease inhibitor, Ov-API-1/Ov-33 [22]; microfilariae surface-associated protein, Ov-MSA-1/Ov103 [23]; and the cysteine proteinase inhibitor, Ov-CPI-1/Ov10/OC 9.3/Ov7 [24],[25],[26]. For each antigen, synthetic DNA optimized for mammalian codon usage was constructed (GenScript Corp, Piscatawy, N.J) for the full-length protein minus the amino acid residues for the signal sequence. Specifically, the fusion proteins used for Ov-Far-1, Ov-API-1, Ov-MSA-1 and Ov-CPI-1 were derived from amino acids 18-178, 18-235, 18-158 and 54-162, respectively, of full-length proteins. Additional details of the nucleotide and amino acid sequences can be found in the GenBank database with accession numbers FJ561736, FJ561737, FJ561735, and FJ561734 for Ov-FAR-1, Ov-API-1, Ov-MSA-1, and Ov-CPI-1, respectively. The four *Ov* templates were amplified by PCR with gene specific linker-primer adapters. In each case, the cDNA fragments were subcloned downstream of Ruc, and a stop codon was included directly after the protein fragment of interest. The PCR primer sequences that were used to generate each construct are as follows: Ov-FAR-1, 5′-GAGGGATCCAACGTGGTGCCCTTCTCC-3′ and 5′-GAGCTCGAGTCAGTTCTT CTGCAGAAA-3′; Ov-API-1, 5′-GAGGGATCCGGAG TGGTGAAGAGATAC-3′ and 5′-GAGCTCGAGTCAGTAGATGGCCACGCA3′; Ov-MSA-1, 5′-GAGGGATCCGACCTGCT GTCAGAGGCC-3′ and 5′-GAGCTCGAGTCAT T CCCTCAGAGTATT-3′; and Ov-CPI-1, 5′-GAGGGATCCGGCTGGGAGGATAGAGAC-3′ and 5′-GAGCTCGAGTCACACCTC CTTTGTGCC-3′. The integrity of all the plasmid constructs was confirmed by DNA sequencing.

### LIPS analysis

LIPS tests were performed in a blinded manner on a large number of sera (see Table 1) at room temperature using a 96-well plate format [27]. First, a “master plate” was constructed by diluting patient sera 1∶10 in assay buffer A (20 mM Tris, pH 7.5, 150 mM NaCl, 5 mM MgCl_2_, 1% Triton X-100) in a 96-well polypropylene microtitration plate. To evaluate antibody titers by LIPS, 40 µl of buffer A, 10 µl of diluted human sera (1 µl equivalent), and 50 µl of 1×10^7^ light units (LU) of Ruc-antigen Cos1 cell extract, diluted in buffer A, were added to each well of a polypropylene plate and incubated for 1 hour at room temperature. Next, 7 µl of a 30% suspension of Ultralink protein A/G beads (Pierce Biotechnology, Rockford, IL) in PBS was added to the bottom of each well of a 96-well filter HTS plate (Millipore, Bedford, MA). The 100-µl antigen-antibody reaction mixture was then transferred to this filter plate and incubated for 1 hour at room temperature on a rotary shaker. The protein bound to the protein A/G beads was washed using a vacuum manifold. After the final wash, LU were measured in a Berthold LB 960 Centro microplate luminometer (Berthold Technologies, Bad Wilbad, Germany) using coelenterazine substrate mix (Promega, Madison,WI). All the LU data shown represent the average of two independent experiments and were corrected for background LU values of the beads incubated with Ruc-antigen extract but without sera. An input of 10 million LU for each antigen was used in both the individual tests and with the antigen mixture format.

### QLIPS

A modified shortened version of LIPS designated QLIPS (for quick LIPS) was also employed [17]. In these assays, patient sera, processed in a 96-well format, were combined with a 4-antigen mixture and buffer for only 5 minutes and then incubated for another 5 minutes with the protein A/G beads. The plates were then washed and LU measured with the microplate luminometer as described above.

### Statistical analysis

**Figure 1:**
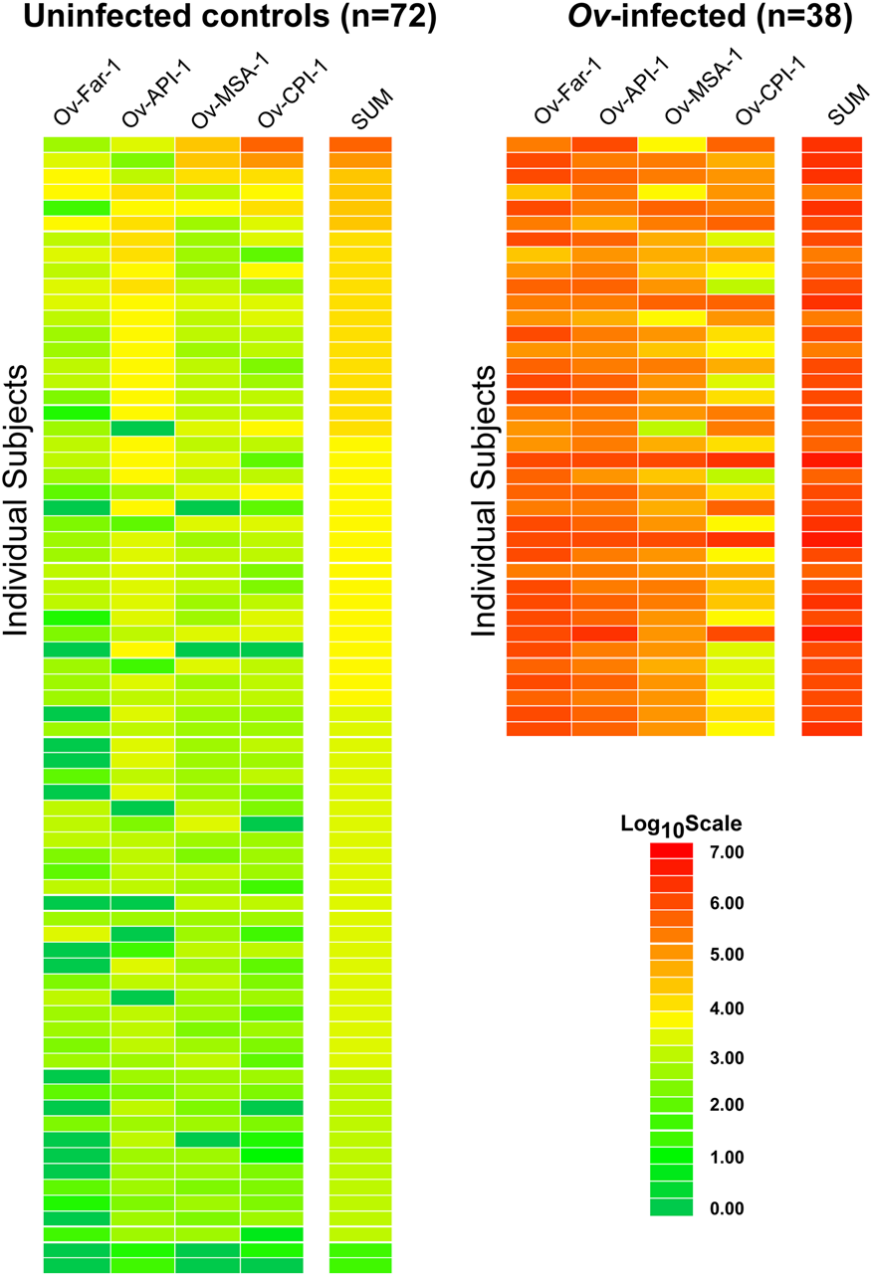
Heat map representation of patient antibody profiles to the 4 *Ov* antigens. The antibody levels for each serum were log_10_ transformed and then the levels were color-coded as indicated by the log_10_ scale on the left, in which signal intensities range from red to green indicating high (red) and low (green) titers. The samples were rank ordered from highest to lowest based on the sum of the antibody titers to the 4 antigen panel. The samples on the left are from uninfected, while the samples on the right are *Ov*-infected sera.

The GraphPad Prism software (San Diego, CA) was used for statistical analyses, including evaluating test performance by area under the curve (AUC) [28]. Results for quantitative antibody levels between the uninfected controls (CTRL), *Ov*-infected, *Wb*-infected, *Ll*-infected and other helminth, (*Ss*)-infected serum samples are reported as the geometric mean titer (GMT)±95% confidence interval (due to the typically overdispersed nature of these data; i.e., they are not normally distributed). Correlations among antibody responses to the four antigens tested were assessed by the Spearman rank test (*r_S_*). The level of statistical significance for all tests was set at *P*<0.05. For determining the cut-off limits for each test two general methods were used. First, cutoff values were derived from the mean value of the 72 uninfected control samples plus 5 standard deviations (SD) and are indicated in the figures. The second method, involved using cut-off values from the two extremes of the receiver operating characteristics (ROC) curve –those being tests that had 100% sensitivity and those tests with 100% specificity using typically highly cross-reactive *Wb*-infected sera as the discriminator. ROC analysis originates from signal detection theory as a model of how well a receiver is able to detect a signal in the presence of noise. Its key feature is the distinction between a true positive rate (sensitivity) and a false positive rate (specificity), been widely used in medical applications to study the effect of varying the threshold on the numerical outcome of a diagnostic test. The non-parametric Mann-Whitney *U* test was used for comparison of antibody titers in different groups and the area under the receiver operator curve (AUC) was used a global index of diagnostic accuracy [29],[30]. Odds ratios, 95% confidential intervals and *p*-values were calculated using logistical regression methods in order to determine the risk for having *Ov*-infection using the cut-off values. For the heat map in Figure 1, the antibody level for each serum was log_10_ transformed and then the levels were color-coded as indicated by the log_10_ scale in the figure legend.

## Results

### LIPS profiling of antibodies to four *Ov* antigens

**Figure 2:**
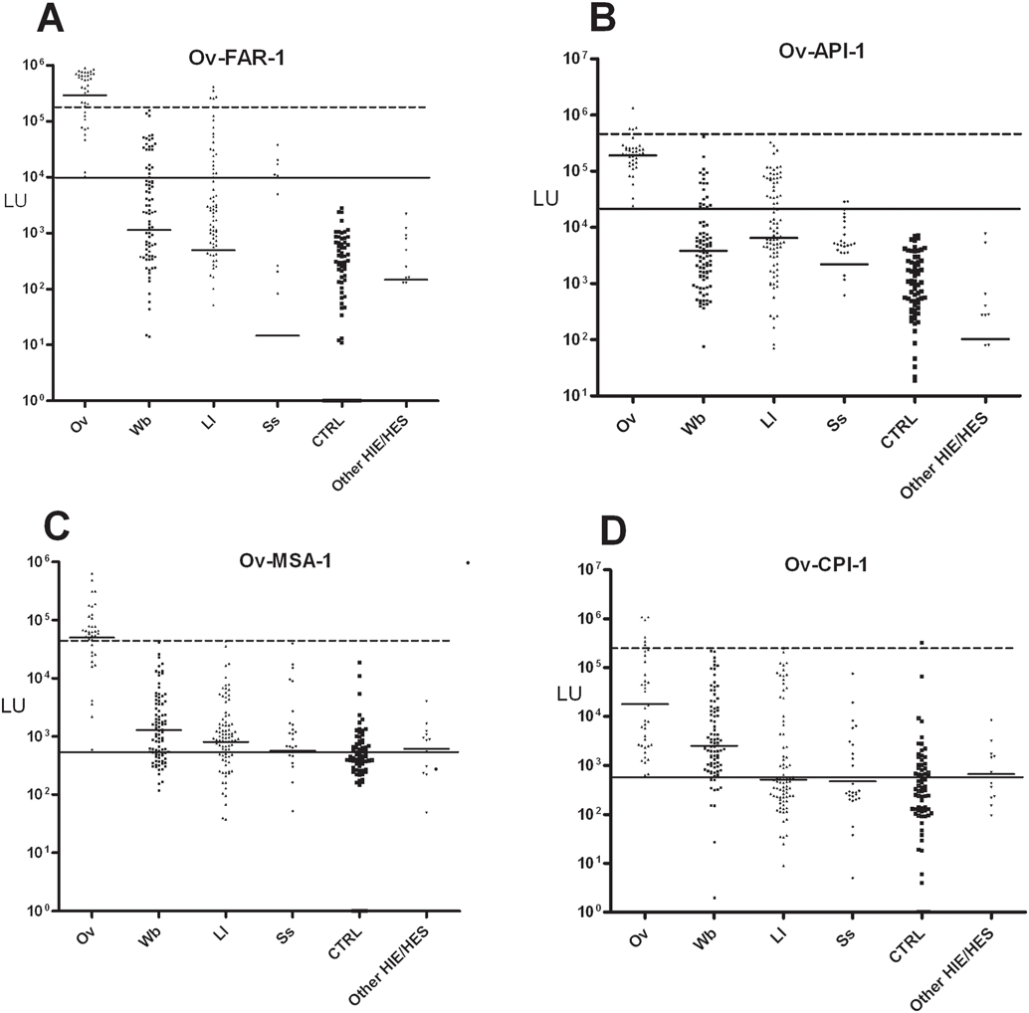
LIPS detection of antibodies to 4 different *Ov* antigens. Each symbol represents individual samples from the 38 *Ov*-infected, 90 *Wb*, 90 *Ll*, 27 *Ss*, 72 control uninfected samples and 12 other control patients. Antibody levels in LU are plotted on the Y-axis using a log_10_ scale and short solid horizontal lines indicate the geometric mean titer (GMT) for each antibody per group. The diagnostic performance related to cross-reactivity with other filarial infections was also evaluated. As described in the text, the long solid line represents the cut-off level corresponding to 100% sensitivity, while the long stippled line corresponds to the cut-off for 100% specificity with sera from the *Wb* cohort.

Previous studies from many different laboratories have identified a large number of potential *Ov* antigens that might be useful for serological screening [8]. The coding sequence for four of the five most promising *Ov* antigens, Ov-Far-1 (Ov-20), Ov-API-1 (Ov-33), Ov-MSA-1 (Ov103) and Ov-CPI-1 (Ov10), were synthetically optimized for mammalian codon usage, constructed as C-terminal fusion with *Ruc*, sequence-verified, and have GenBank accessions numbers FJ561736, FJ561737, FJ561735, and FJ561734, respectively. Evaluation of the utility of LIPS with these four potentially useful antigens began by testing a small number of *Ov*-positive and *Ov*-negative sera samples (data not shown). This panel of four recombinant *Ov* antigens was then tested by LIPS with a large cohort of blinded sera that included patients with parasitologically proven *Ov* infection, other filarial infections, strongyloidiasis, and uninfected controls. Following unmasking, we first determined the utility of these LIPS assays for distinguishing the uninfected CTRL sera from the *Ov*-positive sera. A heat map, employing log_10_-transformed antibody titer data, was used to evaluate these differing antibody responses toward the four *Ov* antigen panel in the 38 *Ov*-infected sera compared to the 72 uninfected CTRL samples (Figure 1). In addition, the sum of the antibody titers from the four individual tests was calculated and is also shown as part of the heat map. As seen in Figure 1, the anti-Ov-FAR-1 and anti-Ov-API-1 antibody tests, as well as the combined data from the four individual antibody tests, clearly distinguished the 38 *Ov*-infected sera from the 72 uninfected CTRL sera. The other two antibody tests, for Ov-MSA-1 and Ov-CPI-1, were also useful but missed several of the *Ov*-positive sera. Furthermore, the anti-Ov-CPI-1 antibody test detected a few weak false positives in CTRL sera (Figure 1, top of heat map). The results of these tests suggest that each of the four *Ov* antigens is highly useful in the LIPS format for distinguishing *Ov*-infected sera from uninfected control sera. Analysis of the GMT for each of the four *Ov* antibody tests revealed that the *Ov*-infected sera had 80- to 4,000-fold higher antibody titers compared to the uninfected control sera. For example, in normal uninfected CTRL sera the GMT for Ov-FAR-1, Ov-API-1, Ov-MSA-1 and Ov-CPI-1 were 78 (95% CI, 42–147); 546 (95% CI, 327–914); 357 (95% CI, 232–549); and 273 (95% CI, 161–464) LU, respectively, whereas the GMT for Ov-FAR-1, Ov-API-1, Ov-MSA-1 and Ov-CPI-1 in the *Ov*-infected sera were markedly higher, with values of 288,713 (95% CI, 196,963–423,203); 190,748 (95% CI, 149,588–243,233); 49,930 (95% CI, 31,402–79,389); and 17,898 (95% CI, 8528–37563) LU, respectively (Figure 2).

Though not obvious from the heat map, further analysis showed that the anti-Ov-FAR-1, anti-Ov-API-1, anti-Ov-MSA-1 and Ov-CPI-1 antibody titers correlated poorly with each other. Our analysis showed that the antibody levels for the four different antigens in the *Ov*-infected positive sera showed little correlation with each other (ranging from *r_S_* = −0.11 to 0.58) suggesting marked heterogeneity in humoral responses of the *Ov*-infected individuals to an individual antigen (Table 2). These results probably reflecting individual differences in MHC Class II haplotypes [31],[32] and suggest that using multiple antigens may be more diagnostically useful than using one or two *Ov* antigens.

**Table 2:** Correlation of antibody titers between the four *Ov* antigens with the 38 *Ov*-infected sera.

Comparisons	*r_s_*	*P* value
Ov-FAR-1 vs. Ov-API-1	0.58	<0.001*
Ov-FAR-1 vs. Ov-MSA-1	0.58	<0.001*
Ov-FAR-1 vs. Ov-CPI-1	−0.11	0.532
Ov-API-1 vs. Ov-MSA-1	0.31	0.058
Ov-API-1 vs. Ov-CPI-1	0.02	0.889
Ov-MSA-1 vs. Ov CPI-1	0.27	0.103

*: *P*<0.05 (significant).

To assess the diagnostic utility of these tests further, the sensitivity and specificity was determined for each of these four tests. For these calculations, the cut-off values derived from the mean plus 5 SD of the 72 uninfected control samples were used for Ov-FAR-1, Ov-API-1, Ov-MSA-1 and Ov-CPI-1 with values of 3,267; 13,727; 10,902; and 196,961 LU, respectively. Using these cut-offs, the Ov-FAR-1 and Ov-API antibody tests showed 100% sensitivity and 100% specificity in distinguishing the 38 *Ov*-infected sera from the 72 uninfected control sera (calculations not shown, but can be derived from Figure 2). ROC analysis confirmed that both the Ov-FAR-1 and Ov-API-1 were perfect diagnostic tests for distinguishing *Ov*-infected from uninfected sera with AUC values of 1.0. The anti-Ov-MSA-1 and anti-Ov-CPI-1 antibody tests performed less well, and showed 89% sensitivity (34/38) with 99% specificity (71/72) and 32% sensitivity (12/38) with 99% (71/72) specificity, respectively (calculations not shown, but can be derived from Figure 2).

### Distinguishing *O. volvulus*-infected sera from other filarial and parasitic infections with the four different antigens

Because of the close phylogenetic relationship between *Ov* and other filarial species and the geographic overlap of *Ov* infections with co-endemic lymphatic filariasis and/or loiasis, as well as with strongyloidiasis (known to have some cross reaction in other serologic assays), additional sera from 90 *Wb*-, 90 *Ll*-, 27 *Ss*- and 12 HIE/HES/other parasite-infected sera were analyzed. As shown in Figure 2, the antibody titer data for other filarial and intestinal nematode-infected sera were plotted alongside the data from the *Ov*-infected samples and uninfected CTRL sera that were previously shown in Figure 1. For the anti-Ov-FAR-1 antibody test, the *Ov*-infected sera showed a more than 250-fold higher GMT of 288,713 (95% CI, 196,963–423203) LU than the *Wb*-, *Ll*- and *Ss*-infected sera (see Table 3), with GMT values of 1,136 (95% CI, 588–2196); 496 (95% CI, 213–1158); and 15 (95% CI, 3–74) LU, respectively (Figure 2 and Table 3). Similarly, the anti-Ov-API-1 antibody test showed a GMT of 190,748 LU (95% CI, 149,588–243,233), in the *Ov*-infected sera compared to the *Wb*-, *Ll*- and *Ss*-infected sera, with GMTs of 3,786 (95% CI, 2545–5633); 6,455 (95% CI, 3723–11,169); and 2,189 (95% CI, 692–6925) LU, respectively (Figure 2). In the case of Ov-MSA-1, the *Ov*-infected sera had a GMT of 49,930 (95% CI, 31,402–79,389) LU compared to the *Wb*-, *Ll*- and *Ss*-infected sera with GMTs of 1282 (95% CI, 932–1765); 803 (95% CI, 545–1181); and 568 (95% CI, 194–1664) LU, respectively (Figure 2). As shown in Table 3, significant differences between the GMT of the *Ov*-infected and the *Wb*-, *Ll*-and *Ss*-infected sera were found for all three antigens (*P*<0.001; Mann Whitney *U* test) as well as for the uninfected control sera.

**Table 3:** Antibody titer characteristics for the *Ov* antigens.

Antigen	Sera	GMT	(95% CI)	*P^a^*	AUC*
Ov-FAR-1	*Ov*	288,713	(196,963–423,203)		
Ov-FAR-1	*Wb*	1136	(588–2196)	<0.0001	0.98
Ov-FAR-1	*Ll*	496	(213–1158)	<0.0001	0.96
Ov-FAR-1	*Ss*	13	(3–64)	<0.0001	0.99
Ov-FAR-1	CTRL	78	(42–147)	<0.0001	1.00
Ov-FAR-1	Other	147	(30–727)	<0.0001	1.00
Ov-API-1	*Ov*	190,748	(149,588– 243,233)		
Ov-API-1	*Wb*	3786	(2545–5633)	<0.0001	0.97
Ov-API-1	*Ll*	6455	(3723–11189)	<0.0001	0.94
Ov-API-1	*Ss*	2189	(692–6925)	<0.0001	1.00
Ov-API-1	CTRL	546	(327–914)	<0.0001	1.00
Ov-API-1	Others	103	(14–745)	<0.0001	1.00
Ov-MSA-1	*Ov*	49,930	(31,402–79,389)		
Ov-MSA-1	*Wb*	1282	(932–1765)	<0.0001	0.96
Ov-MSA-1	*Ll*	803	(545–1181)	<0.0001	0.96
Ov-MSA-1	*Ss*	568	(194–1664)	<0.0001	0.96
Ov-MSA-1	CTRL	357	(232–549)	<0.0001	0.99
Ov-MSA-1	Others	610	(294–1269)	<0.0001	0.98
Ov-CPI-1	*Ov*	17,898	(8528– 37,563)		
Ov-CPI-1	*Wb*	2540	(1465–4404)	0.0002	0.71
Ov-CPI-1	*Ll*	514	(267–992)	<0.0001	0.93
Ov-CPI-1	*Ss*	474	(186–1210)	<0.0001	0.84
Ov-CPI-1	CTRL	273	(161–464)	<0.0001	0.87
Ov-CPI-1	Others	670	(284–1577)	<0.0001	0.89

*P* ^a^ –For the comparisons between the antibody titers in the *Ov*-infected sera with each of the other sera groups by Mann Whitney U test.

*: AUC-area under the receiver operator curve was used as a global index of diagnostic accuracy [29],[30].

The least useful antigen, Ov-CPI-1, showed a GMT in the *Ov*-infected sera of 17,898 (95% CI, 8528–37,563) LU compared to GMTs of the *Wb*-, *Ll*- and *Ss*-infected sera with values of 2,540 (95% CI, 1465–4404); 514 (95% CI, 267–992); and 474 (95% CI, 186–1210) LU, respectively (Figure 2). A significant difference (*P*<0.001; Mann Whitney *U* test) between the anti-Ov-CPI-1 antibody titer was still seen in the *Ov*-infected sera compared to the *Wb*-, *Ll*-and *Ss*-infected sera (Table 3). Finally, very low antibody titers, similar to those of the uninfected CTRL samples were observed in the 14 other (HIE/HES/other) sera for all four of the antigens (Figure 2 and Table 3).

To evaluate the diagnostic specificity of the different antibody tests for detecting *Ov*-infection compared to the other related filarial infections, we used cut-off values derived from the two extremes of the ROC curve: *Ov* antibody tests that were 100% sensitive or 100% specific. For each of these two cut-offs, the *Wb*-infected sera were used as the negative control group (discriminator) because these sera are from geographic areas absolutely without the potential for having *Ov* infection and were likely to be potentially among the most cross reactive. The most informative test, detecting anti-Ov-FAR-1 antibodies with a low end cut-off of 10,393 LU, showed 100% sensitivity and yielded 76% (68/90), 74% (71/90), 82% (22/27), and 100% (72/72) specificity with the *Wb*-, *Ll*-, *Ss*-infected sera and the CTRL sera, respectively (Figure 2A, solid horizontal line). In contrast, a high end cut-off of 156,123 LU for Ov-FAR-1 had 74% (28/38) sensitivity for detecting *Ov*-infected sera but markedly higher specificity values of 100% (90/90), 94% (85/90), 100% (27/27), and 100% (72/72) specificity with the *Wb*-, *Ll*-, *Ss*-infected sera and the CTRL sera, respectively (Figure 2A, dashed horizontal line). In the case, of Ov-API-1, only the low end cut-off had practical diagnostic value. For Ov-API-1, the low end cut-off of 23,945 LU showed 100% (38/38) sensitivity in detecting *Ov*-infected sera with specificity values of 83% (75/90), 68% (61/90), 93% (26/27) and 100% (72/72) with the *Wb*-, *Ll*-, *Ss*-infected sera and the CTRL sera, respectively (Figure 2B). For Ov-MSA-1, only the high end cut-off (41,700 LU) was diagnostically useful and showed 71% sensitivity (27/38) in detecting *Ov*-infected sera with specificity values of 99% (89/90) with *Ll* and 100% with the *Wb*- (90/90), *Ss*- (27/27) infected sera, and CTRL (72/72) sera (Figure 2C). While the anti-Ov-CPI-1 antibody tests showed markedly higher GMT in the *Ov*-infected sera, this test had relatively little practical diagnostic utility when these strict cut-off criteria were used (Figure 2D). In summary, these results suggest that each of the four *Ov* antigens showed preferential immunoreactivity with *Ov*-infected sera compared to the other filarial and intestinal nematode-infected sera. For all four Ov assays, the LU values of the other HIE/HES sera were all similar to or less than the CTRL sera (Table 3).

### Antibody responses to a mixture of four *O. volvulus* antigens

**Figure 3:**
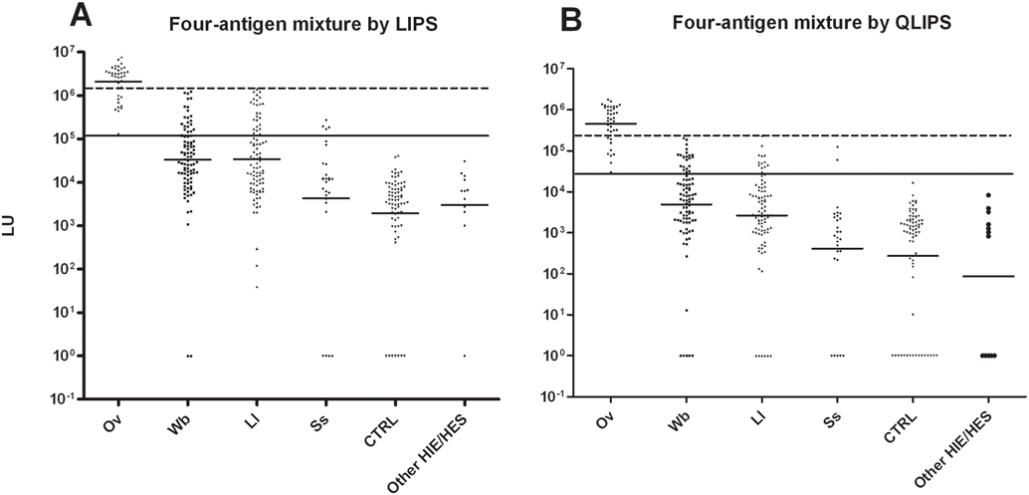
A four *Ov* antigen panel used in the standard 2 hour or QLIPS format shows 100% sensitivity and 100% specificity. Each symbol represents individual samples from the 38 *Ov*-infected, 90 *Wb*, 90 *Ll*, 27 *Ss*, 72 control uninfected samples and 12 other control patients. These LIPS tests were either evaluated as a mixture in the standard format (A) or with QLIPS (B). As shown, both tests showed 100% sensitivity and 100% specificity in distinguishing the uninfected from the *Ov*-infected sera. The diagnostic performance related to cross-reactivity with other filarial infections was also evaluated. As described in the text, the long solid line represents the cut-off level corresponding to 100% sensitivity, while the long stippled line corresponds to the cut-off for 100% specificity with sera from the *Wb* cohort.

Having previously shown that combining two different *Ss* antigens had improved performance over the individual tests [16], we evaluated the diagnostic performance of using a four-*Ov* antigen mixture to assess antibody levels in the same *Ov*-, *Wb*-, *Ll*-, *Ss*-infected sera, CTRL sera, and other conditions (HIE/HES) tested by the individual assays (Figure 3A). The results of the four-antigen mixture showed a GMT for the *Ov*-infected sera of 2,107,000 (95% CI, 1,557,000–2,851,000) LU, which was over 1,000 times higher than the GMT of 1,938 (95% CI, 1,032–3639) LU for the CTRL uninfected sera. Additionally, the GMTs of the antigen mixture with the other infected sera showed much lower values of 33,232 (95% CI, 20,875–52,902); 33,843 (95% CI, 21,887–52,331); 4,307 (95% CI, 962–19,275) and 2,993(95% CI, 541–16,556) LU for the *Wb*-, *Ll*-, *Ss*-infected and the other HIE/HES sera, respectively (Figure 3A). The GMT to the cocktail in the *Ov*-infected individuals were statistically significantly higher than those of the uninfected and other filariasis- or strongyloidiasis-infected sera (*P*<0.0001). Finally, the values from the four-antigen mixture also closely correlated with the sum of the values from the four separate tests (*r_S_* = 0.93, *P*<0.00001) suggesting that in the LIPS format the resulting antibody titer values are highly reproducible under these two different assay conditions.

Cut-off values were next derived from the two extremes of the ROC curve and employed to evaluate the diagnostic performance of this four-antigen cocktail test. With the low end cut-off of 132,785 LU, the four-antigen mixture test showed 100% sensitivity and yielded 78% (70/90), 73% (66/90), 85% (23/27), 100% (72/72) and 100% (12/12) specificity with the *Wb*-, *Ll*-, *Ss*-infected sera and the CTRL and other (HIE/HES) sera, respectively (Figure 3A, solid line). The high end cut-off of 1.2 million LU showed 74% (28/38) sensitivity and had 100% specificity with all the different sera categories (Figure 3A, dashed line). Based on this performance, these results suggest that this four-antigen mixture format is a simple and useful test for the diagnosis of *Ov* infection and for distinguishing most of the *Ov*-infected sera from other filarial and non-filarial helminth infected samples.

### Rapid QLIPS testing using a four antigen mixture

Previously we have used a single antigen in a quick LIPS format (QLIPS), in which the two incubation steps of 1 hour each are reduced to 5 minutes each to assess *Ll* infection more efficiently [17]. Here we tested the diagnostic performance of the QLIPS format using the mixture of four *Ov* antigens with the different infected and uninfected sera. As shown in Figure 3B, the GMT using the QLIPS format was 460,337 (95% CI, 325,109–651,814) LU for *Ov*-infected sera, which was 1,000-fold higher than the GMT of the CTRL sera of 273 (95% CI, 128–580) LU. Compared to the *Ov*-infected sera, other non-*Ov* filarial-infected sera and sera from those with strongyloidiasis showed much lower GMT values of 4,898 (95% CI, 2804–8558) for *Wb*; 2,656 (95% CI, 1517–4648) for *Ll* and 413 (95% CI, 120–1416) for *Ss*. It should be noted that while the overall GMT values from the QLIPS format were approximately 10-fold lower with each of the different sera sub-groups than that with the standard format (compare Figure 3A and 3B), the overall pattern of immunoreactivity of the two assay formats tracked each other well. Nevertheless, with the QLIPS format many more of the non-*Ov*-infected sera showed antibody values of zero (Figure 3B).

With the low end cut-off of 30,000 LU, QLIPS testing showed 100% sensitivity and yielded 76% (68/90), 84% (76/90), 93% (25/27), 100% (72/72), and 100% (12/12) specificity with the *Wb*-, *Ll*-, *Ss*- infected, CTRL, and other (HIE/HES) sera, respectively (Figure 3B, solid line and Table 4). From the high end cut-off of 203,199 LU, the QLIPS format showed 79% (30/38) sensitivity and had 100% specificity with all the sera categories (Figure 3B, dashed line and Table 4). Odd ratios and 95% CI were calculated in order to determine the relative risk for having Ov-infection at a given cut-off. Using a cut-off of 30,000 LU with the QLIPS format, the odds ratio for *Ov*-infected versus *Wb*-infected was 249 (95% CI, 15–4,227) and even higher, 406 (95% CI, 24–6,998), for *Ll*. Thus the presence of antibody titers above 30,000 LU in the QLIPS provides not only a perfect test to distinguish *Ov*-infected from uninfected sera (100% sensitivity and 100% specificity), but also showed relatively high specificity for discriminating *Ov* infection from other often cross-reactive filarial infections (Table 4). For comparison, the cut-off of 132,785 LU in the standard 2-hour LIPS format yielded a similar odds ratio for *Ov*-infected versus *Wb*-infected of 265 (95% CI, 16–4,504) and had an odds ratio value of 209 (95% CI, 12–3,537) for *Ll*. As shown in Table 4, the presence of antibody titers above the cut-off in the standard 2-hour format had a slightly lower performance than that of the QLIPS format for the *Ll*- and *Ss*-infected sera. These results suggest that the QLIPS format using this four-antigen mixture is as good as or slightly better than the four-antigen mixture used in the standard format for distinguishing *Ov* from other filariases and strongyloidiasis.

**Table 4:** Sensitivity, specificity, and odds ratio for the four-antigen mixture determined by LIPS and QLIPS.

Comparisons	Sensitivity	Specificity	Odds ratio (95% CI)	*P* value
**LIPS>132,785**				
*Ov* vs CTRL	100%	100%		
*Ov* vs *Wb*	100%	78%	265 (16–4,504)	<0.0001
*Ov* vs *Ll*	100%	73%	209 (12–3,537)	<0.0001
*Ov* vs *Ss*	100%	85%	402 (20–7,816)	<0.0001
**QLIPS>30,000**				
*Ov* vs CTRL	100%	100%		
*Ov* vs *Wb*	100%	76%	249 (15–4,227)	<0.0001
*Ov* vs *Ll*	100%	84%	406 (24–6,998)	<0.0001
*Ov* vs *Ss*	100%	93%	785 (36–17,060	<0.0001

## Discussion

This study demonstrates that LIPS can detect *O. volvulus* infection by antibody profiling with high diagnostic sensitivity and specificity using a panel of four antigens. The high-throughput, partially automated LIPS and QLIPS formats used here make these approaches highly feasible for screening large numbers of serum samples and/or for rapid field testing. While these *Ov* antigens have been used individually and in combinations as cocktails for screening purposes in ELISA formats, the LIPS tests offered many advantages over conventional ELISA and Western blotting. Some of these advantages include the simple and rapid production of antigens, detection of a large dynamic range of antibody titers without dilution of samples, and the rapid format of the assays that provide superior performance. The robust detection of all four antigens in the LIPS format is likely attributable to the detection of more conformational-specific epitopes. Interestingly, another known recombinant *Ov* antigen, Ov-16 [7] that has performed well in both diagnostic [13] and field-based [12] assays was not immunogenic in the LIPS format likely due to protein misfolding in mammalian cells. Nevertheless, based on the performance of these four *Ov* antigens, it is likely that further modifications of these LIPS tests, such as the addition of new *Ov* antigens, the leaving out of potentially less useful antigens (e.g. Ov-CPI-1), and the adjusting of cut-off values may further simplify testing and improve assay performance.

Similar to our previous published results with *S. stercoralis* [16] and *L. loa* [17], all four *Ov* antigens tested here showed a similar dynamic range in *O. volvulus*-positive sera and relatively similar background binding values in uninfected control sera. It should be noted that several of the *Ov* antigens used here have homologs in other filariae. For example, the 162 amino acid Ov-FAR-1 fragment has approximately 80% similarity with related proteins from *Brugia malayi*, *Wuchereria bancrofti* (*Wb*), and *Loa loa* [33]. Nevertheless, the ability of using these antigens to preferentially distinguish *Ov* infection from other related filarial infections is consistent with other reports [24],[34] and may be further enhanced in the LIPS format due to the detection of many more conformational epitopes. In detecting onchocerciasis, the most informative antigen was Ov-FAR-1, followed by Ov-API-1, Ov-MSA-1 and lastly Ov-CPI-1. The ability to use an antigen mixture in the standard LIPS format or QLIPS format is very convenient as it simplifies data collection and analysis. Furthermore, the values obtained using the sum of the four individual *Ov* tests and those using the antigen mixture closely matched each other, suggesting that the detected antibody titers are highly reproducible regardless of LIPS assay conditions used. In general, increasing the number of antigens within a mixture is generally a favorable condition if each member antigen has a comparable low background signal. In contrast, ELISAs perform poorly when more than one antigen is immobilized and the alternative is to employ complicated multi-epitope hybrid molecules from different *Ov* antigens [14],[15]. Luminex technology can also be used in screening panels of antigens for diagnosis, but require much more expensive equipment and thus has little value for detection of onchocerciasis in most non-hospital or research settings. Consistent with previous studies successfully using cocktails of *Ov* antigens for serological diagnosis [9],[10],[11], the positive results obtained here by LIPS with the four *Ov* antigen mixture supports our previous findings that mixtures of antigens provide more discrimination between infected and uninfected individuals irrespective of the infection being tested [16],[35]. Thus, this general approach of antigen cocktails is likely to be useful for the diagnosis of many other infections.

The QLIPS format performed in less than 15 minutes per 94 samples showed equally promising results for the diagnosis of *Ov*-infection as the standard 2.5-hour LIPS test. Compared to the standard LIPS format, QLIPS showed an approximately ten-fold drop in overall signal in the serum samples but with no change in sensitivity and specificity. In this QLIPS format, all of the *Ov*-infected sera showed positive signals compared to the uninfected sera resulting in 100% sensitivity and 100% specificity. The increased specificity of the QLIPS format over the standard LIPS format for distinguishing *Ll*- and *Ss*-infected sera may be related to the failure of potential low affinity antibodies from individuals with related (non-*Onchocerca*) infections to bind as readily during this short incubation period resulting in much lower signals for these potentially cross-reactive sera. Since we did not test all possible combinations of antigens in the QLIPS format, it is also possible that other antigen combinations might improve performance. As more genomic information is integrated with antigen screening, new immunogenic *Ov* antigens may be identified that might be unique to *O. volvulus*. If such antigens are identified, the incorporation of these proteins may markedly improve the specificity of these LIPS tests. Though not detailed here, the application of LIPS for quantitative analysis of serial antibody responses to a panel of *Ov* antigens could potentially be useful for monitoring response to drug therapy as seen previously in another infection [16] and for sub-stratifying patient subtypes [27].

Compared to other assays formats LIPS showed increased sensitivity and can be performed much more quickly. For example, a field-applicable diagnostic card immunoassay based on a recombinant antigen, Ov-16, showed 80% sensitivity for detecting *Ov*-infected sera [12],[13], while an ELISA employing a recombinant hybrid *Ov* protein showed 93% sensitivity [14],[15]. In these and other studies, issues related to cross-reactivity with other filarial-infected sera such as with *Wb* and *Ll* were not studied with a large enough group of samples and generally showed poorer discrimination of *Ov*-infected sera from these other infections. While the Ov-16 card assay can be performed quickly, other conventional ELISAs require 5–24 hours for completion. In addition to experimental methodology used here, additional LIPS assay formats including microtiter well formats with protein A/G-coated microtiters or gravity filter devices could be utilized. These assays could be performed very inexpensively and be could be employed under less than ideal laboratory conditions.

Our results suggest that the four antigen LIPS/QLIPS has great potential as a tool for early monitoring of changes in the transmission of *O. volvulus* and the recrudescence of onchocerciasis. Like the IgG4-based Ov16 ELISA that is already in use [5], the four-antigen LIPS/QLIPS increases the sensitivity of the assay to 100% without loss of specificity. Such measures will enhance the antibody screening methodology and could provide additional levels of confidence when decisions about cessation of control measures are being made.
